# Regulated Cell Death as a Therapeutic Target for Novel Antifungal Peptides and Biologics

**DOI:** 10.1155/2018/5473817

**Published:** 2018-04-26

**Authors:** Michael R. Yeaman, Sabrina Büttner, Karin Thevissen

**Affiliations:** ^1^Division of Molecular Medicine, Los Angeles County Harbor-UCLA Medical Center, Torrance, CA 90502, USA; ^2^Division of Infectious Diseases, Los Angeles County Harbor-UCLA Medical Center, Torrance, CA 90502, USA; ^3^Los Angeles Biomedical Research Institute at Harbor-UCLA Medical Center, Torrance, CA 90502, USA; ^4^The David Geffen School of Medicine at UCLA, Los Angeles, CA 90024, USA; ^5^Department of Molecular Biosciences, The Wenner-Gren Institute, Stockholm University, Stockholm, Sweden; ^6^Institute of Molecular Biosciences, University of Graz, Graz, Austria; ^7^Centre of Microbial and Plant Genetics (CMPG), KU Leuven, Leuven, Belgium

## Abstract

The rise of microbial pathogens refractory to conventional antibiotics represents one of the most urgent and global public health concerns for the 21st century. Emergence of *Candida auris* isolates and the persistence of invasive mold infections that resist existing treatment and cause severe illness has underscored the threat of drug-resistant fungal infections. To meet these growing challenges, mechanistically novel agents and strategies are needed that surpass the conventional fungistatic or fungicidal drug actions. Host defense peptides have long been misunderstood as indiscriminant membrane detergents. However, evidence gathered over the past decade clearly points to their sophisticated and selective mechanisms of action, including exploiting regulated cell death pathways of their target pathogens. Such peptides perturb transmembrane potential and mitochondrial energetics, inducing phosphatidylserine accessibility and metacaspase activation in fungi. These mechanisms are often multimodal, affording target pathogens fewer resistance options as compared to traditional small molecule drugs. Here, recent advances in the field are examined regarding regulated cell death subroutines as potential therapeutic targets for innovative anti-infective peptides against pathogenic fungi. Furthering knowledge of protective host defense peptide interactions with target pathogens is key to advancing and applying novel prophylactic and therapeutic countermeasures to fungal resistance and pathogenesis.

## 1. Significance of Fungal Infections

### 1.1. Medical Burden of Fungal Infection

In the last two decades, *Candida* species have emerged as the third most common pathogen of nosocomial septicemia, accounting for 5–10% of all hospital-acquired bloodstream infections [1–3]. Overall incidence of candidemia now surpasses incidences of bacteremia due to *Escherichia coli* or *Klebsiella* species [4, 5]. Furthermore, *Candida* species are the most common cause of deep-seated fungal infections in patients who have extensive burns [6] or have undergone transplantation [7–9]. Additionally, *Candida* species are among the most common causes of catheter-related fungal infections [10]. Despite modest advances in antifungal therapy, attributable mortality of candidemia remains approximately 40% [11]. The emergence of highly antifungal-resistant species such as *C. auris* compounds these concerns [12]. Likewise, life-threatening infections caused by *Aspergillus*, *Rhizopus*, and *Mucor* species are seen with increasing incidence as the numbers of patients having immunosuppression in settings of hematopoietic or solid organ transplant, cytotoxic cancer chemotherapy, and related conditions increase [13, 14]. Collectively, these fungal infections have unacceptably high mortality rates that may exceed 50%, even with gold-standard antifungal therapy.

### 1.2. Urgent Need for Innovative Solutions

Convergence of increasing populations at risk of serious fungal infections, emerging resistance to conventional antifungal agents, and a paucity of development of mechanistically novel antifungal therapeutics portends a significant public health concern.

#### 1.2.1. Burgeoning Populations at Risk

Populations of individuals at risk of severe fungal infections are rapidly expanding in scope and number worldwide and are related to several trends with respect to the global population demographics, including the following:

*(1) Aging*. Immune waning and senescence have significant negative impacts on host defense against infection [15, 16]. Infection is now the attributable cause of mortality in nearly one-third of all individuals aged 65 years or older [17]. Estimates project that by 2050, the number of persons ≥ 65 years of age will reach 1.5 billion, and those aged ≥ 80 years will reach 395 million [18, 19]. Thus, along with risk factors imposed by age-related comorbidities, the growing cohort of aging individuals portends significant increases in opportunistic and pathogenic fungal infections.

*(2) Cancer*. Infection is a significant risk factor for morbidity in cancer patients and is a leading cause of attributable death in malignancy [16]. Beyond systemic impacts due to cancer itself, cytotoxic chemotherapy renders patients functionally immunosuppressed, thereby affecting key cell-mediated effectors such as granulocytes and macrophages [20]. Further, hospitalization, long-term in-dwelling catheterization, and chronic exposure to antibiotics enhance their risks of fungal infection [21]. Projections by the World Health Organization and others estimate a dramatic increase in cancer incidence and prevalence in the next two decades [22].

*(3) Surgery*. With other healthcare-associated risk factors, infections due to surgical procedures are rising in incidence and associated with worse outcomes [23]. Moreover, obesity, diabetes, smoking, hypertension, coronary artery disease, and chronic obstructive pulmonary disease [24] are pre and postsurgical risks of infection. Compounding this issue is the wide use of broad-spectrum antibiotic prophylaxis in relation to surgery, which together with immune suppression can increase risks for opportunistic fungal infections.

*(4) Transplant*. Successful transplantation almost always requires prolonged or even life-long immune suppressive therapy. It follows that infectious complications remain a leading cause of morbidity and mortality in settings of organ or hematologic transplantation [25]. Opportunistic fungal pathogens are among the most dangerous etiologies of these infections and can emerge when the host is rendered immunocompromised by regimens necessary for engraftment. In part due to the rise of antifungal resistance, opportunistic fungal infections are increasingly common in transplantation [26].

*(5) Autoimmunity*. Autoimmune diseases affect 5–10% of the population worldwide [27], translating to hundreds of millions of lives impacted. Autoimmune diseases are burgeoning globally [28], and as a group are equivalent in prevalence to heart disease, and twice that of cancer [29, 30]. Immune-modifying therapies used to treat autoimmune diseases have significant risks for life-threatening infection, including those caused by opportunistic and pathogenic fungi [31, 32].

#### 1.2.2. Resistance to Existing Antimycotics

Fungal infections are often difficult to treat for several reasons: (1) few selective targets in these eukaryotic pathogens as compared to the human host; (2) increasing resistance to conventional antifungal agents which have advanced little in the past 50 years; and (3) the lack of mechanistically novel antifungal agents developed for clinical application. As a result, an additional concern is the emergence of resistance to antifungal agents, which are commonly used to prevent and treat disseminated candidiasis. Azole-resistant *Candida* strains are being isolated with increasing frequency, even in patients without AIDS [1, 2]. Although a limited number of antifungal drugs have been developed, experience with antibacterial agents predicts that resistance to novel antifungal drugs will emerge as their use increases [33]. Multiple factors have led to the continuous increase of reported antifungal resistance in the laboratory and in clinical failures due to resistance in human infection. For example, the fact that with few exceptions, the antifungal agents used in clinical medicine today are largely the same as those used for the prior several decades. Thus, as is the case for antibacterial resistance, exposing countless generations of fungal organisms to such agents necessarily affords a survival advantage to those pathogens capable of resistance. Key themes of concern include *Candida parapsilosis*, *C. krusei*, and *C. glabrata* clinical isolates that routinely exhibit reduced susceptibility to multiple antifungal agents [12]. While perhaps less common in incidence than infections due to *C. albicans*, these fungal pathogens can often represent serious or life-threatening bloodstream and invasive infections. Moreover, the advent of pan-resistant species of *Candida*, such as *C. auris*, is among the most significant public health concerns related to fungal infections in recent memory [12]. In addition, infections due to opportunistic or pathogenic molds remain an urgent issue. Infections caused by *Aspergillus*, *Rhizopus*, *Mucor*, and related molds are angioinvasive and destructive, often leading to necrotic and irreversible tissue damage. Infections caused by these Mucoromycotina, and those due to *Cryptococcus*, are typically treated with cytotoxic levels of amphotericin B and typically resist less toxic azoles or echinocandins [33]. Echinocandins are the newest class of antifungals introduced for human therapy in the last decade. However, recent development of echinocandin resistance has been reported via point mutations in *FKS* genes that encode the echinocandin targets (reviewed in [34]), supporting the urgent need to identify novel antifungal agents.

#### 1.2.3. Paucity of Antifungal Development

Amphotericin B was originally discovered in the 1950s, and has been used clinical to treat fungal infections for approximately 50 years. Over that same span of time, remarkable few mechanistically novel antifungal compounds have reached regulatory approval for clinical use. These include 5-flucytosine (1963), the azoles fluconazole (1990) and voriconazole (2002), and the echinocandin caspofungin (2003), posoconazole (2006), and anidulafungin (2007) [33]. Thus, the pharmacopeia of approved antifungal agents for bloodstream or other invasive infections is essentially encompassed by just three mechanistic classes: polyenes, azoles, and echinocandins. While echinocandins are technically considered to have cyclic-peptide-like structures, the scope of the discussion herein focuses on noncyclic host defense and synthetic peptide compounds. Likewise, the many factors contributing to a significant reduction of industry investment in developing novel antifungal agents is beyond the scope of this discussion. Suffice it to say there is now an urgent need to discover and develop structurally and mechanistically new antifungal agents to meet the growing threat of life-threatening infections due to these pathogens.

## 2. Regulated Cell Death in Fungi

An important new area of discovery with great potential to reveal conceptually as well as mechanistically novel targets for next-generation antifungal therapeutics is regulated cell death (RCD), previously referred to as programmed cell death (PCD). For the last two decades, the occurrence of RCD in unicellular eukaryotes has been a topic of intensive study, and fungi have been shown to succumb to several distinct subroutines of regulated cell death. The majority of these studies have been carried out in the well-established model system *Saccharomyces cerevisiae*. According to the classification of cell death modalities suggested by the Nomenclature Committee on Cell Death 2018 and the recent guidelines for cell death nomenclature in yeast [35, 36], *S. cerevisiae* and likely other fungi can undergo at least three distinct types of RCD: intrinsic apoptosis (formerly **type I PCD**), autophagic cell death (formerly **type II PCD**), and regulated necrosis (formerly **type III PCD**). While considerable efforts have historically been focused on gaining a greater mechanistic and biological understanding of these RCD pathways in *S. cerevisiae*, recent studies have also provided new insights into RCD in human pathogenic fungi, including *Candida* and *Aspergillus* species. Here, we summarize the basic concepts and significant advances in this regard.

### 2.1. Biological Roles of Regulated Cell Death in Fungi

The signals, mechanisms, and pathways through which eukaryotic cells functionally age, wane, and ultimately meet death is equally important to their origin, development, and maturation. Thus, RCD has evolved as a means for populations of cells to most efficiently—and least disruptively—censor those individual cells that either do not actively contribute to the larger microbial community or that detract from it. Example settings in which RCD is particularly meaningful in this regard include reproduction, establishment of colonies, quorum sensing and metastatic dissemination from existing colonies or abscesses, biofilm maturation and survival in the face of antifungal agents, and many other processes necessary for survival and pathogenesis. Thus, there is sound rationale supporting fungal RCD as an innovative and mechanistically novel target of next generation antifungal therapeutics.

While the sense of an apoptotic program in unicellular organisms might not be as obvious as in metazoans, emerging data depict scenarios in which the death of damaged or old cells favors the survival of a clonal population [37]. RCD has been shown to occur during physiological scenarios such as unsuccessful mating [38], differentiation of a yeast colony [39], or replicative and chronological aging [40–42]. While replicative aging refers to the number of cell divisions an individual mother cell can undergo, chronological aging reflects the time nondividing cells stay viable in stationary phase. In both aging scenarios, apoptosis serves the elimination of cells that may be old, irreversibly injured, or which have dysfunctions that are detrimental to the larger fungal cell population. Aside from this, the apoptotic program can also be hijacked by competing fungal populations, for example via secretion of killer toxins that trigger cell death in susceptible strains [43].

In aggregate, the RCD subroutines can be induced by a plethora of different stimuli and scenarios, ranging from signaling molecules necessary for survival and reproduction to existing environmental and host threats, including antifungal agents. The relative dependence on distinct molecular players and events for activation of specific RCD pathways appears to be subject to conditional contexts in which the organism encounters a specific stress signal [44]. Based on their molecular and biochemical characteristics, the apoptotic mode of cell death has been subdivided into extrinsic and intrinsic apoptosis [35]. While extrinsic apoptosis is initiated by activation of specific transmembrane receptors that subsequently trigger cellular demise, intrinsic apoptosis can be induced by a variety of cellular stresses that all converge on mitochondria-mediated cell death processes. While yeast does most probably not succumb to the typical extrinsic apoptosis, intrinsic apoptosis does exist and represents the best studied RCD scenario in yeast so far. Morphologically, this mode of cell death is characterized by hallmark features, including systematic DNA fragmentation, nuclear condensation, exposure of phosphatidylserine at the outer leaflet of the plasma membrane, and accumulation of reactive oxygen species.

The fundamental molecular machinery governing apoptotic cell death appears to be evolutionary conserved, and numerous counterparts of mammalian key effectors of apoptosis have been identified in yeast in the past two decades [45–47]. For instance, these include orthologues of the apoptosis-inducing factor (Aif1) and Endonuclease G (Nuc1). When released from mitochondria upon an apoptotic insult and translocation to the nucleus, they cause DNA fragmentation and subsequent cell death [48, 49]. For Endonuclease G, this process involves the mitochondrial adenine translocator, implying the formation and actuation of a mitochondrial permeability transition pore in yeast apoptosis [48].

Mitochondrial features such as hyperpolarization, oxidative burst and generation of reactive oxygen species (ROS), dissipation of mitochondrial transmembrane potential, release of cytochrome *C*, loss of cytochrome *C* oxidase activity, or mitochondrial fragmentation have all been shown to contribute to apoptotic death to varying extents depending on respective triggers and scenarios [50]. Even so, variations on these themes have been observed among differing fungal organisms, including opportunistic yeast and pathogenic fungi, as discussed below.

### 2.2. Regulated Cell Death Subroutines in *Saccharomyces cerevisiae*

Although *S. cerevisiae* is not a typical human pathogen, the emergence of opportunistic *S. cerevisiae* infections has been reported in patients with chronic disease, cancer, and immunosuppression [51]. Moreover, considerable insights into RCD have been gained through studies using this organism as a model. The following discussion considers the genetic and mechanistic aspects of RCD in *S. cerevisiae* in this light, particularly given many of the RCD determinants and pathways have homologous systems in high priority human pathogens, including *Candida* species and pathogenic molds.

#### 2.2.1. Intrinsic Apoptosis (*Type I* PCD)

Intrinsic apoptosis is perhaps the most well-known of RCD pathways, through which cellular constituents are systematically degraded by caspase-dependent or -independent mechanisms. Comparable to mammalian intrinsic apoptosis, apoptosis in yeast is accompanied by release of cytochrome *C* from mitochondria, thus impairing oxidative phosphorylation. However, the participation of cytosolic cytochrome *C* in the formation of apoptosomes, as is observed in mammalian cells, has not yet been demonstrated in yeast.

To date, one orthologue of mammalian caspases has been identified in *S. cerevisiae*: the metacaspase Yca1 (alias Mca1) [52]. Despite different cleavage specificities of caspases (targeting aspartate-X dyads) and metacaspases (targeting lysine-X or arginine-X dyads), these proteases share a common evolutionary origin and are integral to cell death execution. Notably, the protein TSN (Tudor staphylococcal nuclease) has been established as the first common substrate of caspases and metacaspases, arguing for stringent functional conservation despite phylogenetic distance [53]. Approximately 40% of the apoptotic scenarios investigated in *S. cerevisiae* to date depend at least in part on the presence of Yca1 as executor of cellular destruction [54]. The fact that caspase-like activities have been detected even in cells lacking Yca1 indicates that additional proteases with caspase-like activity contribute to RCD in yeast [55]. One such protease may be the separin Esp1, a highly conserved protease that facilitates sister chromatid separation during metaphase to anaphase transition via cleavage of Scc1 (alias Mcd1), a subunit of the cohesion complex [56]. Esp1 belongs to the CD clan (superfamily) of cysteine proteases, a group of proteases that also includes caspases, transamidases, and bacterial gingipains [56]. During hydrogen peroxide-induced apoptosis, cleavage of Scc1 by Esp1 yields a C-terminal Scc1-fragment that relocalizes from the nucleus to the mitochondria, causing the dissipation of mitochondrial transmembrane potential, cytochrome *C* release, and cell death [57]. Several additional proteases have been associated with yeast apoptosis. Among these, the HtrA2/Omi-orthologous serine protease Nma111 is involved in oxidative stress-induced cell death [58] and responsible for the proapoptotic cleavage of the yeast inhibitor-of-apoptosis protein Bir1 [59]. Likewise, the serine carboxypeptidase Kex1 is involved in cell death execution upon treatment with hypochlorite and acetic acid as well as defects in N-glycosylation [60, 61].

Numerous endogenous and exogenous triggers have been reported to initiate intrinsic apoptotic cell death pathways in yeast, through metacaspase-dependent as well as metacaspase-independent pathways [45–47]. Among the endogenous triggers are for instance DNA damage and replication stress [62], defects in N-glycosylation [61], chronological or replicative aging [41, 42], perturbations in cytoskeleton dynamics [63], and impaired mRNA stability [64]. Likewise, a diverse set of exogenous stimuli for yeast apoptosis has been identified, including treatment with low doses of acetic acid and hydrogen peroxide, hyperosmotic stress, heat, high salt concentrations, UV irradiation, heavy metals such as iron, copper, and manganese, ceramide, amiodarone, aspirin, diverse antitumor agents, and many more [45, 46, 65]. Relevant to pathogenesis, specific host defense factors have been shown to act via induction of the intrinsic pathway of apoptosis in various fungi, including *S. cerevisiae*. For instance, peptides from the dermaseptin family produced by amphibians trigger Aif1-dependent apoptosis [66], and the plant-derived defense peptide osmotin kills by activation of Ras-dependent apoptosis [67]. Interestingly, this mode of cell death requires the binding of osmotin to the plasma membrane receptor Pho36 and might thus constitute a variant of extrinsic apoptosis (also see Section (4), below).

Homologues of mammalian proteins integral to the mitochondrial pathway of intrinsic apoptosis have been shown to be functional in yeast and to act through conserved cell death machinery. For instance, heterologous expression of the human proapoptotic protein Bax in yeast causes outer mitochondrial membrane permeabilization, cytochrome *C* release, and cell death [45]. Simultaneous heterologous expression of the human antiapoptotic regulators Bcl-2 or Bcl-XL prevents Bax-induced death and enhances yeast resistance to the apoptotic stimuli H_2_O_2_ and acetic acid [50]. Thus, the intrinsic pathway of apoptosis in yeast can be complemented by human or other mammalian homologues, again highlighting the functional conservation of the apoptotic program.

#### 2.2.2. Autophagic Cell Death (*Type II* PCD)

Autophagy represents the degradation pathway in eukaryotic cells through which the bulk of intracellular molecular turnover occurs, including breakdown of a wide range of cytoplasmic material such as aggregated proteins, organelles, and in some cases, pathogen determinants. During macroautophagy, cargo destined for degradation is sequestered into double-membraned vesicles termed autophagosomes and targeted to the vacuole for subsequent degradation and recycling. Discovered more than half a century ago, autophagy is now recognized as a catabolic process required for the coordinated regulation of cell development, infection control, aging, and other physiological and pathophysiological fates, including cell death. Similar to other RCD subroutines, the fundamental autophagic mechanisms and molecules are conserved across the evolutionary spectrum from microbes to man. Indeed, most of the autophagy-related (*ATG*) genes and pathways have been identified and characterized in yeast cells under nutrient limiting and other stress conditions [68, 69]. While autophagy mostly represents a prosurvival and longevity-ensuring program, excessive autophagy can also contribute to cell death, in particular during development [70]. Autophagic cell death is characterized by increased numbers of autophagosomes along with aberrant protein and organelle turnover. Evidence suggests that autophagic cell death occurs in yeast cells, but the precise contribution of distinct autophagic processes to cell death execution is relatively unexplored [46]. Even so, data supporting this concept are emerging. Heterologous expression of mammalian p53 in yeast causes cell death accompanied by an upregulation of autophagy, and deletion of *ATG1* and *ATG5* partly restores survival [71]. In addition, selective leucine starvation causes a mode of death that requires the presence of essential autophagy regulators such as Atg8 [72]. Indications for an involvement of excessive autophagy targeting mitochondria (mitophagy) derive from studies showing that cells lacking the mitophagy regulator Uth1 are no longer susceptible to cell death induced by expression of mammalian Bax [73]. However, as Bax also causes mitochondria-mediated apoptosis in yeast, the precise contributions of mitophagy to Bax-mediated yeast cell death requires further investigation.

#### 2.2.3. Regulated Necrosis (*Type III* PCD)

Historically, necrosis has been predominantly considered to represent a purely coincidental mode of cell death upon extreme, biochemical, immunological, or mechanical insult that results in membrane rupture, swelling of organelles, and indiscriminant spilling of cell content into its surroundings. In recent years, a more specific view of necrosis has emerged, representing a more fine-tuned and regulated mechanism with implications in inflammatory responses in a variety of physiological conditions [74, 75]. In mammalian cells, the cascade of molecular events and signaling pathways that ultimately leads to necrotic RCD often involves proteases such as calpains and cathepsins, as well as the kinases RIP1 and RIP3 [74]. While most of our knowledge of regulated necrosis comes from mammalian systems, studies demonstrating this form of RCD existing in yeast have been published [76]. During chronological aging, a portion of dying cells exhibit typical hallmarks of necrosis, including loss of membrane integrity and nucleocytosolic redistribution of Nhp6A, the yeast counterpart of mammalian high mobility group box-1 protein HMGB1 [77]. This necrotic, age-associated cell death is inhibited by spermidine, a natural polyamine whose levels decline during the aging process. Spermidine inhibits cell death via an epigenetic process that involves histone H3 deacetylation and induction of autophagy. Thus, the ability of distinct pharmacological and genetic interventions to modulate this process argues that necrotic cell death is highly regulated. In this respect, deletion of distinct histone acetyltransferases blocks regulated necrosis as well [77]. Furthermore, peroxisomal function, perturbation of vacuolar function, and pH homeostasis as well as excess palmitoleic acid and elevated levels of free fatty acids have been associated with regulated necrotic cell death [78–82]. In counterbalance, high levels of the vacuolar protease Pep4, the yeast orthologue of mammalian cathepsin D, prevent both apoptotic and necrotic death during yeast chronological aging. Notably, the inhibition of apoptosis requires the proteolytic activity of native Pep4, while the suppression of necrosis is facilitated by the short propeptide version of Pep4 [83].

### 2.3. Regulated Cell Death in *Candida* Species

**Figure 1:**
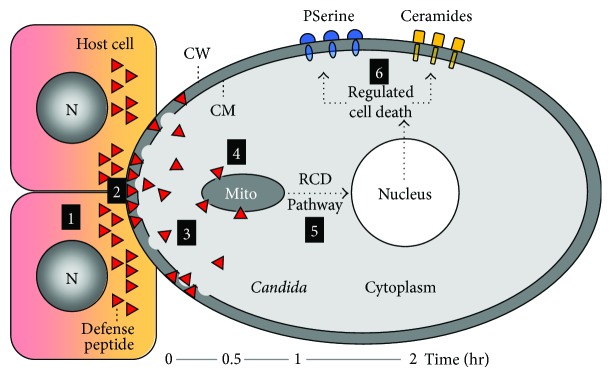
Model of host defense peptide mechanisms versus *C. albicans*. (1) Host cells activated by *C. albicans* deploy prestored and upregulate nuclear- (N-) encoded host defense peptides that directly interact with *C. albicans* to (2) target electronegative cell wall components (e.g., glycosylceramide or all specific cell proteins); (3) permeabilization of the cytoplasmic membrane during or following entry into the cytoplasm; (4) target the electronegative phospholipid composition and transnegative potential (Δ*ψ*) of mitochondria (Mito); (5) perturb mitochondrial functions essential to cell cycle and trafficking, as well as de-energization and respiratory decoupling activation of caspase and/or metacaspase pathway responses; (6) combined effects of cell envelope damage and mitochondrial dysfunction invokes the regulated cell death response which corresponds to hallmark features of apoptosis, including phosphatidylserine (PS) expression. This integrated model is supported by recent publications [115, 125, 131]. It should be understood that different antifungal peptides may exert different mechanisms or a different mechanistic sequence. For example, in the case of plant defensins, the sequence of membrane perturbation and ceramide accumulation has not yet been resolved. It could well be that ceramide accumulation is a first consequence of interaction with glucosylceramides (e.g., step 2; as with RsAFP2). Alternatively, membrane perturbation could potentially be a consequence of the induction of RCD and hence, only appears at step 6.

*Candida albicans* represents the main model system to study fungal pathogenicity and virulence, and RCD of *C. albicans* has been observed upon exposure to various antifungals and other stressors (Figure 1). *Candida* species have been shown to undergo RCD with typical characteristics of intrinsic apoptosis, whereas other forms of RCD such as autophagic cell death and regulated necrosis remain largely unexplored in this organism [84]. Among the triggers for such apoptotic pathways are for instance acetic acid and hydrogen peroxide [85], antifungal peptides from plants [86, 87], and other sources [88, 89], as well as a variety of botanicals such as perillaldehyde, honokiol, baicalin, and cinnamaldehyde [90–93]. In addition, clinical antifungal agents, including caspofungin and micafungin, two echinocandins that disturb cell wall biogenesis, can promote RCD in *C. albicans* [94, 95]. In most of these scenarios, apoptotic death of *Candida* is accompanied by an accumulation of reactive oxygen species and mitochondrial dysfunction [91, 92, 96].

As in *S. cerevisiae*, one metacaspase (Mca1) has been identified in *C. albicans* to date, and Mca1-dependent as well as -independent apoptotic cell death scenarios have been detected [84]. *C. albicans* cells lacking Mca1 are more resistant to oxidative stress-induced cell death than parental organisms [97]. Of note, increased caspase activity has been observed in relation to induction of apoptosis in *C. albicans* exposed to other stimuli, for instance the quorum sensing molecule farnesol [98, 99], the plant-derived macrocyclic bisbibenzyl plagiochin E [96] or silibinin [100]. In contrast, cell death resulting from caspofungin is executed through a Mca1-independent manner but instead requires Aif1 [94, 101].

Amphotericin B, an antifungal agent in clinical use for more than 30 years, triggers apoptotic death of *C. albicans* [85, 102] and inhibits biofilm formation of *C. albicans*, *C. krusei*, and *C. parapsilosis* [103]. Its fungicidal effects are accompanied by increased caspase activities, and concomitant exposure to caspase inhibitors provides cytoprotection. Interestingly, simultaneous pharmacological inhibition of histone deacetylation enhances amphotericin B-induced apoptosis of established *Candida* biofilms [103]. Furthermore, earlier studies connected apoptotic death in *C. albicans* to increased Ras signaling [104] and defects in glutathione synthesis [105]. More recently, human lactoferrin has been reported to induce apoptosis in *C. albicans* via binding and inhibition of the plasma membrane H^+^-ATPase Pma1, which eventually results in disturbed ion homeostasis, mitochondrial dysfunction, and death [106]. Moreover, host cells seem to be able to utilize the apoptotic program of fungal pathogens as a defense system. For example, interaction with macrophages induces metacaspase activation and apoptosis in *C. albicans* [107]. In this process, distinct metacaspase substrates involved in glycolysis and protein quality control were decreased [107].

It should be understood that a given fungal organism may employ distinct apoptotic pathways, depending on multiple factors in context. For example, cell density may affect nutritional availability or activate quorum sensing pathways leading to apoptosis [108]. Thus, the effects of farnesol or other signals with respect to quorum sensing, biofilm formation, and cell death may vary depending on the microenvironmental conditions in context of infection and the strategies of fungal growth characteristics therein (e.g., yeast versus hyphae).

### 2.4. Regulated Cell Death in Pathogenic Molds

Compared to *Saccharomyces* or *Candida* species, less is known regarding RCD in *Aspergillus* species (Ascomycetes) or their Mucoromycotina cousins, *Rhizopus* or *Mucor*. However, recent data point to parallels in RCD among pathogenic fungi. Farnesol-induced quorum-sensing mechanisms may exist in *Aspergillus*, ultimately yielding RCD [109]. For example, in *A. nidulans*, farnesol induces the expression of an apoptosis-inducing factor (AIF-) like mitochondrial oxidoreductase, mitochondrial ATPase inhibitor, and cytochrome *C* peroxidase. As a result, ROS accumulation and mitochondrial fragmentation is observed, consistent with a process of caspase-independent apoptosis in this organism. Early studies also suggest that *Mucor* species have explicit RCD responses to stress. For instance, the HMG-CoA reductase inhibitor lovastatin can inhibit posttranslational modification of proteins, including prenylation. Following exposure to lovastatin, *M. racemosus* arrested sporangiospore germination, yielding profound cytoplasmic condensation and DNA fragmentation [110]. More recent studies reported that specific sesterterpene-type metabolites, including ophiobolins A and B, can induce apoptosis in *Mucor* species [111]. The calcineurin pathway governs key virulence and antifungal resistance pathways in *Rhizopus* as well as in other pathogenic molds such as *Mucor*. Interestingly, when exposed to the calcineurin inhibitor tacrolimus, the fungistatic triazole agents posaconazole and itraconazole became fungicidal for *R. oryzae* [112]. This effect was accompanied by DNA fragmentation, phosphatidylserine externalization, ROS accumulation, and activation of caspase-like functions. From these examples, there is considerable evidence supporting the view that caspase-dependent and -independent pathways of RCD exist in pathogenic molds and yeasts.

## 3. Regulated Cell Death as an Antifungal Peptide Target

Host defense peptides (HDPs) of different structural scaffolds occurring naturally or engineered have now been shown to activate fungal RCD by way of key mechanisms, including perturbation of quorum sensing, mitochondrial function, autophagy or mitophagy, disruption of replication or reproductive mechanisms, and/or interference with cell cycle and aging. A particularly attractive aspect of targeting RCD in developing novel antifungal agents relates to the potential minimization of unintended consequences of inflammation that may accompany death of fungal cells exposed to cytotoxic agents that induce unregulated necrotic death. The following discussion focuses on selected examples from recent studies that offer insights into the RCD-inducing mechanisms of host defense peptides and how they might be exploited as novel antifungal agents and strategies. Recent and prior evidence is considered from studies of RCD mechanisms differentially induced by distinctive classes of antifungal HDPs (summarized in Table 1).

### 3.1. Helical and Extended Peptides

Perhaps the most widely recognized structural class of peptides having antifungal activity are those exhibiting *α*–helical or extended structures. Examples of this group of molecules for which evidence of RCD has been reported are discussed below.

#### 3.1.1. Periplanetasin-2

This peptide has recently been isolated from the cockroach *Periplaneta americana* and studied in terms of its mechanism of action against *C. albicans* [113]. The synthetic amide version of the peptide led to lipid peroxidation, accumulation of ROS, externalization of phosphatidylserine, dissipation of *Δψ*, and loss of cytochrome *C* from mitochondria, with activation of caspases, DNA fragmentation, and eventual cell death. Therefore, by inducing insurmountable oxidative stress, it appears that periplanetasin-2 evokes intrinsic apoptosis in *C. albicans*.

#### 3.1.2. Scolopendin

Scolopendins are recently identified antimicrobial peptides of centipedes [89]. A prototype scolopendin was found to induce apoptosis in *C. albicans*, as evidenced by mitochondrial dysfunction, ROS accumulation, cytochrome *C* release, deenergization, phosphatidylserine externalization, chromatin condensation and fragmentation, and cell death. These RCD mechanisms were associated with metacaspase activation.

#### 3.1.3. Lactoferrin

Lactoferrin is one of the most extensively studied HDPs. In early work, Andres et al. showed that this peptide induces apoptotic cell death in *C. albicans* via K+ channel-mediated K+ efflux [114]. More recently, this same group has shown lactoferrin to induce apoptosis by inhibiting the membrane H+-ATPase Pma1, leading to subsequent mitochondrial dysfunction [106]. Interestingly, Yount et al. previously noted that toxicity of human beta-defensin 2 (h*β*D-2) and crotamine toxin corresponded to common structure-activity relationships promoting targeting of eukaryotic K+ channels, including that of *C. albicans* [115]. Human lactoferrin has also been found to trigger caspase-dependent cell death in *Saccharomyces* [116].

#### 3.1.4. Histatin-5

It remains unclear whether the antifungal peptide histatin-5, found in human saliva and oral secretions, induces apoptosis in *C. albicans*. Investigations by Vylkova et al. showed that histatin-5 evokes osmotic stress responses [117], and ensuing studies by Sun et al. demonstrated it to perturb ATPase functions in *C. albicans* [118]. Prior to those studies, Helmerhorst et al. demonstrated that histatin-5 exerts its antifungal activity through the formation of reactive oxygen species [119] and also showed histatin-5 to target fungal energetic systems and to cause mitochondrial dysfunction [120]. Collectively, such findings strongly implied RCD-like mechanisms. However, studies by Wunder et al. reported that histatin-5 does not exert its antifungal mechanisms via apoptosis [121].

### 3.2. *γ*–Core Peptides

Cysteine-stabilized (CS) HDPs across the phylogenetic continuum share a 12–18 residue multidimensional structure-function feature known as the *γ*–core [115, 122]. While this structure-function signature is conserved across a vast evolutionary distance, accessory sequences have adapted to optimize functions in distinct host anatomic and microbiologic niches. For instance, this fundamental motif is conserved in peptides of the defensin, CS*αβ*, and many other distinct peptide families originating from bacteria, fungi, plants, insects, and humans [123], but the accessory domains have undergone evolutionary radiation. Examples of apoptosis-inducing *γ*–core peptides that have antifungal efficacy are considered below.

#### 3.2.1. Plant Defensins RsAFP2 and HsAFP1

As with all defensin-family polypeptides of plants, RsAFP1, RsAFP2, and HsAFP1 are cysteine-stabilized, cationic peptides containing a multidimensional *γ*–core motif [122, 124]. Plant defensins are in general characterized by broad-spectrum antifungal activity and typically have minimal toxicity to plant or human cells in vitro [125]. Originating from the common radish plant family member *Raphanus sativus* and the coral bell *Heuchera sanguinea*, the respective plant defensins RsAFP2 and HsAFP1 are models for studying apoptotic mechanisms of action versus *C. albicans*. Aerts et al. were perhaps the first to demonstrate that a plant defensin (RsAFP2) can induce intrinsic apoptosis in *C. albicans*, independent of the metacaspase Mca1 [86]. This observation was followed by a report on the induction of apoptosis of *C. albicans* by another plant defensin, HsAFP1 [87]. Interestingly, HsAFP1 was the first plant defensin for which a direct interaction with a fungal-specific lipid-based receptor was demonstrated [126], however at that time, this receptor could not be identified. Later studies by Thevissen and coworkers demonstrated that RsAFP2 interacts with fungal-specific glucosylceramides (GlcCer) [127]. The GlcCer constituents are present in the cell membrane and wall of susceptible fungi. As RsAFP2 cannot interact (or does so to a substantially lesser extent) with structurally distinct GlcCer from plant or human cells [127], the induction of RCD is selective to organisms that contain the fungal-specific sphingolipid receptor. This relationship explains why RsAFP2 is not active against the emerging fungal pathogen *Candida glabrata*, as this organism does not produce GlcCer due to a lack of the *GCS1* gene encoding glucosylceramide synthase [128]. Subsequently, Thevissen et al. demonstrated that RsAFP2 is not internalized in the cell per se, but associates with the cell envelope, resulting in cell wall stress, septin mislocalization, and accumulation of ceramides in *C. albicans* [129]. The latter effects are likely responsible for the induction of apoptosis; however, the exact mechanism yielding RCD relative to the RsAFP2-induced ceramide accumulation is hitherto unknown. Thus, multiple lines of evidence suggest this peptide induces RCD by way of targeting mitochondrial and cell cycle functions.

These antifungal effects of RsAFP2 were also found to be synergistic with caspofungin in mitigating the pathogenic consequences of *C. albicans* biofilms [130]. In the case of HsAFP1, the identity of the fungal-specific membrane receptor has not yet been elucidated. Its fungicidal action exploits the oxidative respiratory chain in *C. albicans* to cause hyperaccumulation of ROS among other phenotypic markers of apoptotic death. Interestingly, genes associated with conferring susceptibility to this peptide were those mediating mitochondrial response and related stress-induced functions [87]. Very recent evidence indicates that HsAFP1 can bind to phosphatidic acid (PA) and to a lesser extent, to various phosphatidyl inositol moieties [131]. Specifically, this peptide accumulates at the cell surface of yeast cells with intact membranes, most notably at the buds and septa, and is subsequently internalized. Further, PA was found to play an influential role in the internalization of HsAFP1, as yeast expressing reduced PA levels internalized less of this peptide (Thevissen, unpublished data). However, additional as yet unknown fungal-specific processes and targets also appear to be playing a role in HsAFP1 internalization. Likewise, in the case of the plant defensin NaD1, cell wall components have been implicated in its internalization [132, 133]. Microbicidal effects resulting from induction of RCD, such as energy perturbation and membrane permeabilization, are presumably secondary effects that ensue following HsAFP1 internalization [131].

#### 3.2.2. Plant Defensin-Like Peptides

The seeds of the leguminous plant *Adenanthera pavonina* are the source of a recently identified peptide that exerts apoptotic mechanisms of action against fungi [134]. This defensin-like plant polypeptide termed ApDef-1 causes cell cycle dysfunction and concomitant intracellular ROS accumulation and chromatin condensation prior to metacaspase activation and cell death by intrinsic apoptosis. It is not yet known if ApDef-1 triggers these effects through general stress responses or if prototypic fungal pathogens such as *C. albicans* or pathogenic molds are susceptible to this mechanism of action. The LpDef1 peptide recently isolated from the seeds of *Lecythis pisonis* causes the accumulation of ROS and mitochondrial dysfunction in *C. albicans*, pointing towards RCD as mechanism of action [135].

#### 3.2.3. Insect Defensin Coprisin

Coprisin is an antimicrobial peptide from the dung beetle with features of the broader insect defensin family [136, 137]. In its 43 amino acid form, there are two Cys-disulfide bonds that stabilize its characteristic CS–*αβ* structure. Lee et al. reported this form of coprisin to exert energy- and salt-dependent mechanisms of RCD in *C. albicans* [138]. These activities include accumulation of intracellular ROS, dysfunctional mitochondrial *Δψ* and cytochrome *C* release, phosphatidylserine externalization, and metacaspase activation, leading to apoptotic death of *C. albicans*.

#### 3.2.4. Fungal Defensin-Like Peptides

Along with higher eukaryotic organisms, fungi themselves appear to have exploited RCD over an evolutionary timespan as a means to protect themselves from other competing fungal organisms. For example, the *Neosartorya fischeri* antifungal protein (NFAP) is a basic, cysteine-rich, extracellular antifungal protein with structural similarity to defensins [139]. This peptide is characterized by a *β*–barrel topology, constituting five highly twisted antiparallel *β*–strands, stabilized by disulfide bridges. However, in contrast to its *β*–defensin relatives, NFAP contains a hydrophobic core [140]. Homologues of this peptide have also been isolated from *Aspergillus giganteus* (*Aspergillus giganteus* antifungal protein (AFP)), *Aspergillus clavatus* (*Aspergillus clavatus* antifungal protein (AcAMP)), and *Aspergillus niger* (*Aspergillus niger* antifungal protein (ANAFP)) [141, 142]. Specifically, NFAP is produced by the *N. fischeri* NRRL 181 isolate (anamorph: *Aspergillus fischerianus*). Heterologous expression of the *nfap* gene in the NFAP-sensitive *A. nidulans* revealed the induction of intrinsic apoptosis, as well as damage and dysfunction of the cell wall, the destruction of chitin filaments, and the accumulation of nuclei at the broken hyphal tips [143].

#### 3.2.5. Human Defensins

Human neutrophil defensin-1 (hNP-1) and human beta-defensin 2 (h*β*D-2) are among the most predominant of the host defense peptides elaborated within neutrophils and expressed by the integument (epithelial barriers), respectively. Notably, h*β*D-2 has been shown to perturb mitochondrial energetics and induce phosphatidylserine accessibility in *C. albicans* [115]. Such results indicate that these peptides exert their candidacidal mechanisms at least in part via a RCD response involving mitochondrial targeting. These effects were influenced by pH and occurred in relationship to altered cell membrane permeability. Similar mechanisms of anticandidal activity have been observed for hNP-1. Extending on these findings, two genes have now been identified in HDP resistance in *C. albicans* and *S. cerevisiae*. Gank et al. showed that the gene *SSD1* is integral to the ability of these fungi to survive in the face of human defensins [144]. Moreover, an *ssd1* null *C. albicans* strain was significantly less virulent in a mouse model of infection as compared to its wild-type counterpart. Subsequent investigations by Jung et al. demonstrated that the regulatory gene *BCR1* also contributes to host defense peptide resistance in *C. albicans* likely via a pathway that intersects that of *SSD1* [145]. Of note, a synthetic *β*–hairpin peptide (RP-13) that lacks a *γ*–core motif did not exert anticandidal activities identical to those of hNP-1 or h*β*D-2.

#### 3.2.6. Kinocidins

Kinocidins are chemokines that exert direct microbicidal activities and potentiate functions of synergistic immune effectors, such as leukocytes [123, 146, 147]. These host defense peptides have common structural configurations comprising three modular domains: (1) N-terminal domain containing the chemokine cysteine motif; (2) interposing *γ*–core domain; and (3) C-terminal microbicidal helical domain [147, 148]. All mammalian kinocidins are characterized by this structural pattern. Human kinocidins representing all four conventional chemokine cysteine array structure groups (C, CC, CXC, and CX_3_C) have been identified and demonstrated to have direct microbicidal activity against human pathogens [147], and congeners of these molecules have been engineered for enhanced antimicrobial activity [149, 150]. The two predominant groups of kinocidins are distinguished as *α* (CXC) or *β* (CC and other). Examples of *α*–kinocidins (CXC) include platelet factor-4 (PF-4; CXCL4) and platelet basic peptide (PBP; CXCL7), interleukin-8 (CXCL8), monokine induced by interferon-*γ* (MIG-9; CXCL9), interferon-γ inducible protein-10 kDa (IP-10; CXCL10), and interferon-inducible T cell α-chemoattractant (I-TAC9; CXCL11) [123, 147, 151]. Specific examples of *β*–kinocidins (CC and other subgroups) include monocyte chemoattractant protein-1 (MCP-1; CCL2), macrophage inflammatory protein-1 (MIP-1; CCL3), RANTES (regulated upon activation, normal T cell expressed/secreted; CCL5), and lymphotactin (CL1). The importance of these concepts and roles for host defense peptides having more than just antimicrobial activity are reviewed elsewhere [146, 152].

Kinocidin holoproteins and their modular peptide domains exert direct antifungal activities which are conditionally dependent. For example, kinocidins CXCL1 (GRO-a), CXCL8 (IL-8), CCL5 (RANTES), and CL1 (lymphotactin) exert significant candidacidal efficacy at pH 5.5 but little or no activity at pH 7.5 in vitro [147]. The fungicidal effect of the CXCL8 holoprotein was observed with as little as 1 nmol/ml, with complete sterilization of a 6 log CFU inoculum of *C. albicans* exposure to 5 nmol/ml of this protein. Importantly, the hemipeptide of CXCL8 containing the *γ*–core and microbicidal helix accounted for all of the antifungal activity of the holoprotein. For example, the isolated microbicidal helix of CXCL8 at 0.5 nmol/ml exerted sterilization of a 6 log inoculum of this organism in the same solution phase assay in vitro. Interestingly, whereas the kinocidins typically exert strong anti-*C. albicans* activity at pH 5.5, their efficacy is considerably less at pH 7.5. This pattern of activity is opposite to that of these peptides versus bacteria, which generally is substantially greater at pH 7.5 than pH 5.5 in vitro.

Mechanisms of kinocidin antifungal action have been studied using *C. albicans* clinical isolates and genetic mutants. For example, a synthetic peptide congener (RP-1) designed on the microbicidal helices of mammalian CXCL4 (PF-4) family kinocidins caused invagination and permeabilization of the cell membrane, condensation of cytoplasmic macromolecules, and loss of mitochondrial energetics in wild-type *C. albicans* [145, 153]. Gank et al. and Jung et al. demonstrated that Ssd1 and Bcr1 proteins function in a pathway that is integral to the survival of this organism in the face of low concentrations RP-1 and other host defense peptides [144, 145]. To gain further mechanistic insights, *C. albicans* wild type, *Δssd1*/Δ*ssd1* null, *SSD1* complemented and forced overexpression mutants were exposed to RP-1 under distinct pH conditions simulating bloodstream (pH 7.5) or abscess (pH 5.5) contexts in vitro. Mechanisms of action were then evaluated using multiparametric flow cytometry assay devised to simultaneously assess six modes of activity (osmotic homeostasis; macromolecular condensation; cell membrane permeability; mitochondrial energetics; phosphatidylserine display; and caspase-like protease activity) [153]. *SSD1* expression inversely correlated with antimicrobial peptide susceptibility, mitochondrial deenergization and phosphatidylserine accessibility. Moreover, *SSD1* expression corresponded to mitigation of membrane permeability (assessed by propidium iodide staining, PI) and caspase-like protease induction and was greater at pH 7.5 than pH 5.5. Collectively, these results suggest that RP-1 and perhaps other host defense peptides induce RCD in *C. albicans* which can be modulated to some extent by Ssd1 protein. Similar studies focusing on *Δbcr1*/Δ*bcr1* mutants of *C. albicans* demonstrated that the Bcr1 protein functions downstream of Ssd1 to mediate low level resistance to RP-1 and perhaps other host defense peptides by fostering homeostatic membrane integrity and mitochondrial energetics in vitro [145]. Furthermore, a homozygous null mutant of *SSD1* (Δ*ssd1*/Δ*ssd1*) was significantly less virulent in a murine model of hematogenous candidiasis [144]. Thus, the Ssd1-mediated pathway also plays an important role in the survival of *C. albicans* during infection in vivo.

### 3.3. Other Regulated Cell Death-Inducing Peptides

A variety of other natural peptides and proteins have been shown to induce apoptosis in yeast. Examples include osmotin [67], melittin [154], the aspergillocidal peptide PAF of *Penicillium chrysogenum* [155], the surfactant protein WH1 fungin produced by *Bacillus amyloliquefaciens* [156], a truncated derivative of dermaseptin S3 [66], yeast pheromone [157], psacotheacin [158], and the killer toxins produced by the yeast *Kluyveromyces lactis* [159]. Other peptides for which evidence exists of apoptosis-related mechanisms of action are reviewed in De Brucker et al. [160]. Additional examples of peptides that exert antifungal activity but for which RCD has not been substantiated are reviewed in [161].

## 4. Development of Peptide Anti-Infectives Targeting Fungal RCD

Meritorious attempts have been made to translate the potent and rapid in vitro antimicrobial activities of naturally occurring host defense and related peptides of higher organisms into novel anti-infective drugs. For a variety of reasons, this goal has proven quite elusive to date. Even so, exciting advances are emerging regarding structure, mechanism, and development of peptide-based agents. In the following discussion, the main challenges experienced to date and opportunities on the horizon are reviewed.

### 4.1. In Vitro to In Vivo Translation

There have been numerous examples of antibacterial peptide efficacy in animal models of infection. However, few of these agents have successfully reached phase III clinical trials, and no host defense peptide isolated from higher organisms or engineered mimetic thereof has achieved regulatory approval for use in clinical therapy [162, 163]. Even less progress has been made with respect to development of peptide therapeutics targeting fungal infections. There are only few reports that document in vivo efficacy of antifungal peptides. In this respect, Tavares et al. demonstrated efficacy of the native plant antifungal peptide RsAFP2 when administered prophylactically in a mouse candidiasis model [164]. Moreover, combined therapy of PAF, the small antifungal protein from *Penicillium chrysogenum*, and amphotericin B (AMB), which act synergistically in vitro, was more effective than either AMB or PAF treatment alone in a mouse model for invasive pulmonary aspergillosis [165]. Aside from toxicologic and pharmacologic barriers (see below), one significant issue in this respect is the reality that in vitro activity does not consistently translate to in vivo efficacy. As discussed above, the challenge of context likely plays a major role in this regard. For example, many published methods assess antimicrobial peptide efficacy in austere buffer systems that lack relevant physiologic constituents or conditions. Complicating this issue is the observation that even the most bioactive peptides can exhibit little or no efficacy in complex media (e.g., Mueller-Hinton or Brain-Heart Infusion broth) often used for standard MIC testing. Thus, the relevance of in vitro susceptibility testing of any antimicrobial peptide should be considered in relation to these limitations. A more relevant but more cost- and labor-intensive method involves testing peptide compounds in blood biomatrices, including whole blood, plasma, and serum ex vivo [166]. This method has two important advances over assays done in buffer systems: (1) peptides must overcome binding to molecular or cellular constituents present in the matrix and (2) additive or synergistic functions of a peptide with other host defense mechanisms can be assessed, for example, potentiation of neutrophil opsonophagocytosis and intracellular killing [146].

Translation of in vitro activity to in vivo efficacy has also been limited with respect to antifungal peptide efficacy. Gank et al. correlated in vitro hypersusceptibility of *C. albicans* to host defense peptides with in vivo hypovirulence in a murine model of hematogenous dissemination [144]. Very recently, Cools et al. demonstrated that truncated HsAFP1-based peptides spanning the *γ*–core were very active in combination with caspofungin against biofilms in vitro. However, translation to in vivo conditions failed due to the interaction of the truncated peptides with serum albumins [167], which could not be resolved by their subsequent PEGylation or cyclization. Interestingly, however, native HsAFP1 still displayed activity in the presence of serum albumins, possibly owing to its structured configuration. Hence, it is plausible that the use of native HDPs or their engineered congeners, alone or in combination with standard antimycotics, bears great potential to combat fungal infections including those resistant to current therapies.

### 4.2. Off-Target Effects and Toxicity

One key challenge to therapeutic development of peptide antifungal agents relates to the undefined toxicity of certain candidates tested clinically to date. Several examples of anti-infective peptide candidates failing in preclinical development or early-stage clinical trials have been cited for reasons largely due to off-target effects and toxicity. Notable cases in this regard have been reviewed elsewhere [151, 168, 169]. As detailed above, evidence strongly supports the concept that some host defense peptides and engineered congeners thereof act at least in part by inducing or eventuating RCD in fungal target cells. This double-edged sword is likely a result of multiple convergent issues: (1) unicellular (e.g., fungi) and multicellular (e.g., mammals) eukaryotes share common molecular machineries and signal-response pathways that are responsible for RCD; (2) evolutionary, structural, and functional parallels exist among families of host defense peptides, venoms, and toxins [115]. For example, reciprocal structure-function relationships exist among human defensins such as hBD-2 and venoms/neurotoxins such as rattlesnake crotamine sharing related *γ*–core structural motifs [115, 170]. Mechanistic data and computational modeling also support such relationships. For example, hBD-2 and crotamine each significantly perturb mitochondrial energetics and induce phosphatidylserine accessibility of *C. albicans* and human endothelial cells in vitro [115]. Likewise, Aarbiou et al. showed that hNP-1 and LL-37 induce death of human lung epithelia and human immortalized T lymphocytes (Jurkat T cells) by mitochondrial injury [171]. In support of these concepts, Zhu et al. reported that mutations of two residues in a core consensus insect defensin sequence possessing a structural signature related to toxogenic effects of scorpion venoms could convert a defensin into a neurotoxin [172]. Similarly, Vriens et al. reported on the mutation of one residue in the sequence of the antifungal plant defensin PDF2.3 from *Arabidopsis thaliana* (AtPDF2.3), thereby converting its partial scorpion toxin signature into the full toxin signature [171–173]. As a consequence, this mutant AtPDF2.3 was characterized by antifungal activity and inhibitory activity toward mammalian Kv1.2 and Kv1.6 potassium channels [173]. Therefore, untoward toxicologic mechanisms and determinants that may reside in host defense peptides chosen to serve as antifungal templates will need to be addressed during preclinical and clinical development.

In balance, there are important opportunities pertaining to the potential for host defense peptides or their mimetics to be developed as novel antifungal agents targeting RCD. For example, crotamine was found to occlude eukaryotic (mammalian K_v_1.2 or *C. albicans* CAK) potassium channels in molecular simulations using NMR structures. However, while hBD-2 engaged them with lower affinity, it did not occlude either of these mammalian or prokaryotic potassium channels (*Escherichia coli* KcsA) [115]. This finding suggests there are definable structure-activity features that may guide the differentiation of antimicrobial activity from host cell toxicities. Likewise, the means by which antifungal peptides or their congeners target cells may be exploited or used to improve selective toxicity. For instance, RsAFP2 has been shown to interact directly with fungal-specific glucosylceramides [127]. Interestingly, while RsAFP2 is not internalized following this interaction, a related antifungal peptide (HsAFP1) does undergo internalization and may induce RCD in target organisms. Moreover, plant defensin NaD1 is unable to permeabilize fungal cell membranes or kill target cells when the *β*-glucan layer is removed, suggesting a specific target or vulnerable process localizes to this layer [133]. Li et al. have reported Ssa1/2 to be a key cell envelope-binding protein for histatin-5 in targeting *C. albicans* [174]. In the case of plant defensin MtDef4, its internalization is energy dependent and mediated through endocytosis in *Neurospora crassa* [175]. Nonetheless, to date there are few other examples of specific receptors for antifungal peptides, and only limited information exists on their exact mechanisms of uptake or selective toxicity.

One notable advance that may facilitate development of antifungal peptide drugs relates to the recent discovery that peptides evolved to target or govern mitochondrial functions in eukaryotic cells contain sequence domains that exert potent antimicrobial activity in vitro [176, 177]. This interesting area is based on the evolutionary progression that led to eukaryotic symbiosis with prokaryotes in the form of mitochondria. Many of these peptides or engineered mimetics thereof induce classic features of RCD in fungi but not in human cell lines. Thus, subtle structural and/or mechanistic features may be designed into peptides to afford greater selective toxicity that targets fungal RCD as an anti-infective strategy. Moreover, particularly advantageous aspects of antifungal peptides that are ultimately developed to target RCD include their likelihood to mitigate a proinflammatory storm of necrotic cell death that may result from antifungal agents inducing survival countermeasures of the target organism.

### 4.3. Pharmacologic Uncertainties

An essential concept has been recognized with respect to naturally occurring antimicrobial peptides, and which has direct implications on the development of antifungal peptide therapeutics: distinct peptides have evolved in context to optimally defend distinct anatomic, physiologic, and microbiologic niches in their host [122, 123, 151]. For example, the human *α*–defensins hNP-1, -2, -3, and -4 are exclusively confined to neutrophil granules. These peptides must be able to function in settings of reduced pH and high levels of reactive oxygen species that are integral to the microbicidal milieu of neutrophil phagolysosomes. By comparison, *α*–defensins HD-5 and HD-5 (alias, cryptdins) are expressed by Paneth cells of small intestine villus crypts. Similarly, human *β*–defensins such as hBD-2 are only predominantly expressed by skin and cutaneous epithelial barrier cells such as keratinocytes. Importantly, while hNPs, HDs, and hBDs share the cysteine array characteristic of the defensin peptide family [170], they differ in sequence and composition as likely optimized to best defend the distinct niches in which they are expressed. It follows that attempts to deploy these peptides or their nonnatural congeners as systemic antimicrobials have largely met with loss of activity or poor efficacy, degradation or lack of durable availability, and/or toxicity when placed out of context.

Defining the conditional optima for peptide antimicrobial activities is an area that remains to be fully explored. Even so, several aspects of the microenvironment in which antimicrobial peptides best function to target pathogens and spare hosts have been studied. These include ion or salt concentrations, pH, protein binding, and growth phase/status of the target organism, among other factors [151, 178, 179]. Lakshminarayanan et al. demonstrated that conditions which alter transmembrane potential and membrane rigidity protect *C. albicans* from lethality due to tetravalent peptide B4010 (4 copies of the sequence RGRKVVRR linked through a branched lysine) [180]. Another challenge to development of peptide anti-infectives is their vulnerability to proteolytic degradation by a variety of proteases encountered systemically, be they en route to or enriched within sites of infection. For example, metalloproteases, trypsin, and chymotrypsin as well other proteases can cleave peptides into nonfunctional fragments. In addition, proteases generated by the pathogens themselves have been demonstrated to serve as resistance mechanisms to certain host defense peptides [181–185].

Various strategies have been used to circumvent the issues of proteolytic degradation that could negatively affect pharmacology of anti-infective peptides. A specific approach that may facilitate therapeutic advancement is the structural design of antifungal peptides or mimetics that retain potent and multimodal mechanisms of action but which are less susceptible or inert to rapid degradation. For example, use of nonnatural amino acids (e.g., D- versus L-enantiomeric amino acids), peptoid platforms and cyclization are methods that have been explored in reducing enzyme vulnerability of lead candidate templates as a means to improve half-life, distribution, and other pharmacokinetic parameters [151, 169]. Notable in this latter regard is the *θ* (theta) family of defensins that are found in leukocytes of Old World monkeys and orangutans, which are cyclic peptides generated by a posttranslational ligation event of two truncated *α*–defensin substrates [186]. Likewise, cyclic orbitide peptides from *Euphorbiaceae* spp. and novel cyclodepsipeptides of fungal organisms represent intriguing antifungal templates [187, 188]. Indeed, naturally occurring peptide-based molecules have illustrated success in this regard and are approved for clinical use as antifungals. For example, echinocandins are peptide-based compounds that inhibit the synthesis of cell wall glucan in fungi including *Candida* spp. and *Cryptococcus neoformans* and often have fungistatic activity against some pathogenic molds (e.g., *Aspergillus*). Members of this antifungal class (caspofungin, micafungin, and anidulafungin) are highly complex cyclic hexapeptoids conjugated to N-linked lipids that competitively inhibit 1,3–*β* glucan synthase [189]. Echinocandin therapeutics are derived from the papulacandin family of naturally occurring antifungals originally isolated from *Papularia* spp. of marine fungi. However, while these agents are technically peptide-based, they are quite distinct from mammalian or plant host defense peptides. Ultrashort peptides, including amidated sequences [190], may offer new insights into antifungal peptide therapeutic agents or strategies. Other approaches include liposomal formulation, continuous infusion, and topical applications.

Opportunities also exist for innovative approaches to development of antifungal peptides based on natural designs and their biological roles in a certain context. For example, specific peptides also have distinct functions under differing conditions and are processed in context to render distinctive functional modules. These themes are established for many kinocidins including CXCL4 and CXCL8, which undergo context-specific cleavage by relevant proteases to yield fully autonomous microbicidal helices [148, 179, 191]. Interestingly, their helical domains exhibit differences in antimicrobial spectra and have greater antifungal activity at pH 5.5 than pH 7.5. Innovative approaches based on such natural processing may afford strategic opportunities to exploit contextually optimized antifungal peptide structures or mechanisms. For example, context-activated protides are synthetic, multimodular propeptides designed to sense and be activated in direct response to strategic microbial or host signals—including proteases and other enzymes—in contexts of infection [123, 192]. Thus, these agents are intended to have complementary advantages with respect to their therapeutic potential. First, they are designed to intensify in the immediate proximity of infection, targeting derivative antimicrobial effector modules to microbes. Second, in contrast to conventional antibiotics which select for resistant pathogens, protides are biased to favor organisms that lack or do not express virulence determinant activators and are poorly or nonpathogenic. Thus, beyond enhanced efficacy and improved targeting of the infecting microbe, context-activated protides have the intriguing potential to shift the evolution of pathogens in favor of nonpathogenic phenotypes. Context-activated protide lead candidates are currently in the process of preclinical development.

Perhaps even more specific to the potential development of biologics targeted for fungal efficacy are recently invented proteins and peptides that induce cell death by exploiting regulated cell death evolutionary relationships [176]. These molecules are designed in part based upon the structure-function relationships of nuclear-encoded proteins that regulate mitochondrial function. As with the context-activated protide platform, the molecules in this technology platform are in the preclinical stage of development.

### 4.4. Sourcing and Manufacturing Issues

Isolation of naturally occurring antimicrobial peptides from native organisms or synthesis of natural or engineered forms of these molecules has historically been seen as time-, labor-, and cost-intensive for feasible commercial development. Therefore, expression systems are most commonly considered for bulk production necessary for commodity-scale use. However, by virtue of their antimicrobial activities, heterologous expression in microbial systems (e.g., *E. coli*) can present issues regarding generation of large amounts of bioactive compound [193]. In this regard, codon-use bias, intracellular granule storage, and misfolding have been among the key pitfalls encountered. To partially circumvent these issues, heterologous expression in the eukaryotes *Pichia pastoris* and *Penicillium chrysogenum* has been proven successful for production of various types of plant defensins [194] and other cysteine-rich, cationic, antifungal peptides [195], respectively. However, the above issues may remain considerable barriers in terms of sourcing and manufacturing of anti-infective peptides, directing production by means other than classical culture and recovery systems. However, recent advances in peptide expression and other innovative approaches including solid-state synthesis have provided new and practical methods to surmount many of these issues. A notable example in this regard was the large-scale heterologous expression of the defensin-family peptide plectasin, from the saprophytic fungus *Pseudoplectania nigrella*. Generation of this cysteine-stabilized peptide at a commodity scale and GMP quality as necessary for human clinical trials provides proof of concept that such peptides can be produced as therapeutics [196]. In addition, the number and diversity of regulatory approved peptide-based therapeutics is growing rapidly, demonstrating how peptides or biologics targeting microbial RCD or other mechanisms can overcome sourcing and manufacturing barriers [197].

## 5. Prospectus

**Figure 2:**
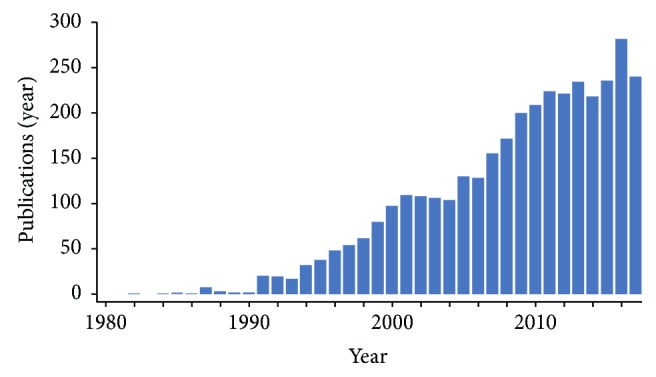
Trajectory of antifungal peptide publications 1980–2017. [Clarivate Analytics accessed Nov 2017].

Host defense peptides from natural sources have long held promise to serve as templates for novel anti-infective agents. This optimism stems from the rapid and potent antimicrobial efficacies of many such compounds, which often exert multimodal mechanisms of action that are considerably less prone to resistance development than static small molecules. The scope of structural and functional features of antifungal peptides has burgeoned in recent years (Figure 2). This trend is expected to accelerate in the coming decade, given the urgency with which novel antifungal and other antimicrobial agents are needed to meet the growing threat of resistance. Many pathogenic fungi are highly refractory to existing anti-infective agents, resulting in high rates of morbidity and mortality. Compounding this challenge are the significant increases in the projected incidence and prevalence of conditions predisposing to invasive fungal infections, including cancer, respiratory diseases such as chronic obstructive pulmonary disease (COPD), organ transplantation, and others. As reviewed herein, of special importance is the opportunity for innovative peptides or mimetics thereof to exploit RCD pathways in fungi as novel therapeutic targets. This strategy has multiple conceptual advantages, including mechanistic novelty and the mitigation of inflammatory storm responses that may occur as fungi attempt to counteract traditional antifungal agents. For example, fungi such as *Candida albicans* may create a systemic and cytokine storm-like effect following the application of antifungal therapy. Likewise, other fungal pathogens may participate in immune reconstitution inflammatory syndromes (IRIS) in the setting of infection in immune compromised hosts. Therefore, controlling fungal infections through regulated cell death has the potential to minimize or avoid these unintended consequences of conventional antifungal therapy.

To realize the potential for evolutionary-proven efficacy of natural antifungal peptides as innovative therapeutics, an intensive and coordinated research and development process is needed, including: (1) host and fungal science to better understand and optimize fungal RCD as a selective target for peptide-based therapeutics; (2) structure-mechanism relationship studies to define the key sequence and/or 3-dimensional determinants of peptides that selectively induce RCD in fungal cell targets; (3) medicinal chemistry to minimize or eliminate off-target host cell toxicity and optimize the pharmacologic delivery of antifungal peptide therapeutics; and (4) feasible and cost-efficient methods for generating commodity-scale peptide anti-infective drugs at GMP quality for clinical use. Achieving these goals appears to be in reach with sustained prioritization of novel antifungal agents to meet the rising tide of fungal infections anticipated in the coming decades.

## Figures and Tables

**Table 1:** Main classes of host defense peptides (HDPs) shown to induce RCD.

Main classes of RCD-inducing antifungal peptides^∗^		
Name	Source	Mode of action apart from RCD induction and mitochondrial dysfunction
A. Helical and extended peptides		
Periplanetasin-2	Cockroach (*Periplaneta americana)*	Lipid peroxidation, caspase activation
Scolopendin	Centipedes Class *Chilopoda*	Metacaspase activation
Lactoferrin	Bovine/human	Metacaspase activation, inhibition of membrane H^+^-ATPase Pma1

B. *γ*–core containing peptides		
Plant defensin RsAFP2	Radish (*Raphanus sativus)*	Interaction with fungal-specific glucosylceramide, induction of cell wall stress, ceramide accumulation, septin mislocalization, metacaspase independent
Plant defensin HsAFP1	Coral bell (*Heuchera sanguinea)*	Interaction with PA and PI phospholipids, accumulation at buds and septa, internalization, pH dependent activity in vitro
Plant defensin-like peptide ApDef-1	*Adenanthera pavonina*	Cell cycle dysfunction, metacaspase activation
Insect defensin Coprisin	Dung beetle Family *Scarabaeoidea*	Dysfunctional mitochondrial Δ*ψ* and cytochrome C release
Fungal defensin-like peptide NFAP	*Neosartorya fischeri*	Cell wall dysfunction, accumulation of nuclei at broken hyphal tips
Fungal defensin-like peptide AFP	*Aspergillus giganteus*	Cell wall perturbation
Neutrophil defensin hNP-1	Human	Membrane permeabilization and depolarization
Beta defensin h*β*D-2	Human	RCD modulated by Bcr1 and Ssd1 proteins in *C. albicans*
Kinocidins (e.g., CXCL4, CXCL8)	Mammalian	Perturb membrane energetics and inhibit macromolecular synthesis; pH-related activity in vitro; RCD modulated by Bcr1 and Ssd1 proteins in *C. albicans*

^∗^Note that Histatin-5 and plant defensin-like peptide LpDef1 were not integrated in the table as their potential induction of RCD is still under investigation.
